# Investigating the role of soil-transmitted helminth infections in the development of leprosy in endemic regions

**DOI:** 10.3389/fimmu.2025.1499304

**Published:** 2025-06-25

**Authors:** Vikram Singh, Ravindra P. Turankar, Vinay Kumar Pathak, Itu Singh, Rahul Sharma, Anjana Goel, Utpal Sengupta

**Affiliations:** ^1^ Stanley Browne Laboratory, The Leprosy Mission (TLM) Community Hospital, New Delhi, India; ^2^ Division of Descriptive Research, Indian Council of Medical Research, New Delhi, India; ^3^ Department of Biotechnology, GLA University, Mathura, Uttar Pradesh, India

**Keywords:** STH infections, leprosy, immunomodulation, IFN-γ, multibacillary leprosy, IL-10, il-12, Th1 & Th2 response

## Abstract

**Background:**

India alone contributes nearly 54% of the global load of new cases of leprosy and 21% to global helminthic parasitic infection cases. Research studies have suggested that Soil-transmitted helminth (STH) infection can regulate the host’s immune response and make them susceptible to leprosy. This study aimed to investigate the association between helminth infection and leprosy.

**Materials & Methods:**

Stool samples (n=360) were collected from 96 patients and 264 household contacts (HHCs) from the endemic region in Purulia (West Bengal) and Champa (Chhattisgarh) India. Samples were examined microscopically for the presence of intestinal helminthic parasites; Cytokine profiling (IFN-γ, IL-12, IL-10) was performed by ELISA on a subset of helminth-positive and negative patients to assess immune responses.

**Results:**

Intestinal parasites were detected in 26% of leprosy patients and 17% of HHCs. Male patients with helminth infection had a significantly higher risk of multibacillary leprosy (OR = 2.60; 95% CI: 1.22–5.55; *p* = 0.019); no significant association was observed in females or overall, between cases and HHCs. IFN-γ levels were significantly reduced in helminth-positive cases (mean 19.70 pg/ml) compared to helminth-negative cases (mean 46.60 pg/ml; *p* < 0.02), indicating Th1 suppression. IL-12 and IL-10 levels did not differ significantly between groups. Over five years, 30 HHCs developed leprosy, but no significant association with baseline helminth status was observed (*p* = 0.816).

**Conclusion:**

Although STH co-infection suppressed Th1 responses in leprosy patients, no direct population-level association with leprosy incidence was established. Integrated parasite control measures may complement leprosy programs by mitigating potential immunomodulatory risks, particularly in high-burden settings.

## Introduction

Leprosy is an infectious disease caused by *Mycobacterium (M). leprae or M. lepromatosis* that primarily affects the skin and peripheral nerves (1, 2). This disease is prevalent in more than 182 countries worldwide, posing a significant health problem, particularly in developing nations. India, for instance, was declared to have achieved the elimination of leprosy as a public health problem in 2005 after attaining the prevalence rate (PR) of <1/10,000 cases set by the World Health Organization (WHO) (3). However, still the country witnessed a 37.7% surge in the detection of new cases in 2022 compared to the previous year, and it still harbours 54% of the world’s leprosy patients (4).

In addition to the above, Asia accounts for nearly 70% of the global burden of soil-transmitted helminths (STH) infection, with the highest prevalence in India (21%) followed by China (18%) (5). The main species that infect humans include *Ascaris lumbricoides*, *Trichuris trichiura*, and *Ancylostoma duodenale*/*Necator americanus*. These worms reside in the host’s intestines and produce numerous eggs daily, which are discharged in the environment like soil and water. Infection most commonly exists in rural communities in warm, humid climates with poor sanitation. Those most affected are usually the economically disadvantaged. The eggs of these helminths which are present in human waste can lead to soil contamination in areas with inadequate sanitation. This implies that people living in these regions with poor sanitation are prone to helminthic infections and are more likely to contract them (6).

Leprosy is manifested in different clinical spectral forms depending on the host’s immune response, which may vary from person to person. While Tuberculoid (TT) or Paucibacillary (PB) leprosy patients show a Th1 immune profile with higher levels of interleukin-2 (IL-2) and interferon-γ (IFN-γ), lepromatous (LL) or multibacillary (MB) patients exhibit a Th2 immune profile with the rise in IL-10 and IL-4 interleukins (7). A study has suggested that co-infection with helminths is associated with the development of multibacillary leprosy. This association may be attributed to the induction of a Th2-biased immune response in the host (8, 9). This type of immune response is characterized by the production of Th2 cytokine (IL-10) typically associated with allergic and humoral immune reactions. Thus, altered immune responses may increase the vulnerability of individuals to MB forms of leprosy (8, 10).

Building on this immunological interaction, it is hypothesized that helminth infections—especially STH—might actively influence host immunity, potentially undermining resistance to *M. leprae.* Various studies indicate that STH infections can downregulate protective Th1 responses, leading to a shift in the immune balance towards a Th2-dominant or humoral response (11–13). This shift could impair macrophage activation and intracellular killing mechanisms, which are crucial for controlling *M. leprae*, thereby increasing vulnerability to more severe, multibacillary forms. While epidemiological data support this association to some extent, direct laboratory evidence is still lacking. Understanding the immunomodulatory role of STH in leprosy is vital, as it may impact clinical manifestations, disease progression, and treatment outcomes. Testing these hypotheses through well-designed *in vitro* or *in vivo* models is essential for elucidating these mechanisms and guiding integrated disease management strategies.

Moreover, recent studies have indicated that co-infection with STH may affect the occurrence of lepra reactions at diagnosis. In endemic regions where both infections coexist, immune reconstitution following deworming might provoke inflammatory episodes due to the halt of chronic helminth-induced immune suppression (14). This finding highlights an essential intersection between parasitic and mycobacterial diseases, underscoring the need for further exploration of the intricate interactions between host immunity, environmental factors, and co-infections.

The present research involved diagnosing STH by examining stool samples under a microscope to detect eggs and larvae, aiming to assess the role of infections in susceptibility to leprosy. The study was carried out in leprosy-endemic areas - the Purulia district of West Bengal and the Champa district of Chhattisgarh, which are known for their high prevalence rates of leprosy [PR of Champa (1.54/10,000) and of Purulia (3.55/10,000] and helminth infections (15). A cohort of 96 leprosy cases and their 264 household contacts (HHC) were followed for over five years at 6-month intervals for the development of leprosy. Additionally, levels of Th1 and Th2 cytokines were measured using enzyme-linked immunosorbent assay (ELISA) was used in leprosy cases (with and without STH parasitic infections) in whole blood culture assays. This study is the first of its kind in India to specifically address the interplay between two major neglected tropical diseases in understanding the susceptibility to leprosy.

## Methodology

### Study subjects’ recruitment

The study included a total of 96 cases of leprosy and 264 of their household contacts (HHCs) who were enrolled after providing informed consent, following the ethical standards and guidelines of the Indian Council of Medical Research and TLMTI Ethical Committee. All leprosy patients, along with their household contacts, were systematically categorized based on sex, age, and specific type of leprosy (as detailed in **Table 1**). This classification underscores the importance of understanding the demographics and variations in leprosy cases for more effective intervention and treatment strategies. All participants were newly diagnosed with untreated leprosy and received care at The Leprosy Mission Trust India in the Purulia district of West Bengal and in the Champa district of Chhattisgarh, India. Qualified physiotherapists assessed all leprosy patients and subsequently confirmed their diagnosis by dermatologists in a TLM hospital in a leprosy-endemic area.

- Voluntary Participation: Prior to participation, patients were adequately explained about the study and provided their consent in an informed manner.
- Community Outreach: The field assistant obtained the addresses of enrolled leprosy patients from the TLM hospital registration desk and subsequently visited their residences to enrol household members in the study. One day prior to sample collection, the field assistant explained the procedure for stool sample collection and provided appropriate containers with labelling code to all participating households. The following morning, both the laboratory technician and the field assistant collected the stool samples and processed them according to standard methods for the laboratory detection of helminthic parasites.

**Table 1:** Demographic details of the leprosy patients and their household contacts who developed leprosy.

Purulia district of West Bengal							
Category	Sex	Total samples number	Mean age (in years) +SD	PB	MB	Chi-square (P value)	Odd ratio
Index cases	Male	48	34.4 ± 14.7	2 (4%)	46 (96%)	0.0001	2.09 (95% CI: 0.17-26.4)
	Female	12	35.1 ± 14.9	1 (8%)	11 (92%)		
HHCs	Male	62	29.3 ± 14.7	3 (5%)*	5 (8%) *	N/A	N/A
	Female	85	29.3 ± 15.2	4 (5%)*	2 (2%) *		
Total number		207					
Champa District of Chhattisgarh							
Category	Sex	Total Samples	Mean age (in years) +SD	PB	MB	Chi-square (P value)	Odd ratio
Index cases	Male	29	34.4 ± 14.7	0	29 (100%)	0.0005	26.82 (95% CI: 1.12 - 644.44)
	Female	7	30.9 ± 15.2	2 (29%)	5 (71%)		
HHCs	Male	54	29.6 ± 15.4	(0) *	8 (15%)	N/A	N/A
	Female	63	29.1 ± 15.2	3 (5%) *	5 (8%) *		
Total number		153					

(*HHCs developed a type (PB & MB) of leprosy after follow-up of 5 years); N/A, not applicable; Significant results are shown in bold (P<0.05); OR (odds ratio); CI (confidence interval).

### Study setting and context

The cohort study was conducted in two leprosy-endemic areas: the Purulia district of West Bengal and the Champa district of Chhattisgarh. The prevalence rate (PR) of leprosy at Purulia was recorded as 3.55/10,000, and that of Champa was recorded as 1.54/10,000 population (16). We selected these sites because leprosy patients and the areas were endemic for both leprosy and STH infection.

• Inclusion criteria

- Confirmed diagnosis of leprosy: We have verified cases of leprosy at TLM Hospital and classified them according to WHO criteria as either PB or MB leprosy.
- Consent form: Patients and their household contacts provided their consent and expressed their willingness to participate in this study and provided their stool samples for detection of helminthic parasite.
- Residing in an endemic area: Patients were selected from areas with known prevalence of leprosy and helminthic infections.
- No recent deworming treatment: At the time of sample collection period, it was confirmed from the ASHA workers regarding whether any recent deworming treatment was done or not in the community. It was noted from the ASHA workers and local authorities of Public Health Centres that there was no such deworming programme was carried out in the community in the recent past.

• Exclusion Criteria

- Patients on recent anti-helminthic treatment
- Severe illness or complications that prevent participation.
- Inability to provide stool samples
- Pregnant or lactating women.

**Time Period**

- We gathered details about new leprosy patients from the medical records at the TLM hospital. The study lasted for three years, and we recruited patients for two of those years.

**Age Range**

- We included a wide range of participants, consisting of both adults and children, with ages ranging from 5 to 75 years. This approach ensures representation of both elderly patients and children in the leprosy community.

### Stool specimen collection

We gathered stool samples the day after commencing multidrug therapy (MDT) for the primary patients and subsequently collected samples from their household contacts living in the same home. The stool samples are collected early in the morning from the patients’ residences. Stool specimens were collected in plastic wide-mouthed 10 ml stool containers and were distributed to all household members in the evening. Before collecting the specimen, the containers were labelled and distributed to the patients with their household name with sample code numbers and collection dates and times. The following morning, the technician collected plastic containers containing stool specimens. These were kept cold on ice in an ice box for transportation to the TLM hospital laboratory. Individuals who failed to provide specimens were reminded in the evening of the preceding subsequent collection days. After three requests, individuals who did not provide the samples were excluded from the study. Each stool sample was examined using the direct saline and iodine wet mount method.

### Processing of stool specimen and direct wet mount analysis

Stool specimens were obtained from all patients and their household contacts enrolled in the study. Microscopically, STH intestinal parasites were detected from stool samples using direct saline and iodine wet mount methods. A small portion of stool samples were preserved in 10% formalin solutions to examine the ova, eggs larva, and the rest of the solution.

Then, preserved specimens were processed using the Formol-Ether concentration method for screening to find the presence of helminth ova and larvae in the sample. The stool samples were examined under a compound microscope at 100 and 400 magnifications as per routine TLM hospital stool examination protocol.

### Formol−ether concentration method

Stool specimens were processed following (17) protocol, with some modifications. Specimens were processed to identify intestinal helminths by placing 1 g of stool in a conical centrifuge tube with 7 ml of 10% formol water. The resulting suspension was filtered, and ether acetate was added to the deposit. The centrifuge tube containing the suspension in ether acetate is shaken vigorously before centrifugation at 3200 rpm for 1 min. After discarding the supernatant, a smear was prepared from the sediment and observed under the microscope 10X and 40X after drying as per routine TLM hospital stool examination protocol.

### Data quality assurance

We followed strict procedures to ensure high data quality. The lab technician and the field assistant carefully collected stool and blood specimens. Specimens were labelled and transported in cold chain boxes. We educated study subjects to prevent contamination. Contaminated specimens were rejected. Direct stool examinations were performed immediately after receipt of the samples in the laboratory and accurate observations were recorded.

### Blood samples collection

The blood sample was collected by applying the tourniquet tightly onto the hand above the elbow to engorge the antecubital vein. The skin above the antecubital vein was sterilized with a 70% ethanol-soaked swab, and then a 2 ml syringe was used for the aspiration of blood by puncturing the vein. Blood samples were transferred into heparinized vials. Peripheral blood samples of 2 ml were aseptically collected from ten leprosy patients with helminthic infection and from nine leprosy patients without helminthic infection. The collected blood samples were transported immediately between 4° - 8°C to the laboratory for whole blood assay for interleukins.

### Whole blood assay for estimation of interleukins

The protocol for the whole blood assay was standardized in Stanley Browne Laboratory following the method of Tran et al. (18) with some modifications. Blood (2 ml) was taken by venipuncture from each subject in a heparinized vial and immediately cultured in a 96-well Nunc (Microwell 96-Well), Nunclon Delta-Treated, Flat-Bottom; Nunc, Denmark; Cat no. 167008) plate for the whole blood assay.

In Brief, the blood was diluted 1:5 with sterile RPMI 1640 (Sigma-Aldrich, USA; Cat no. R5886) tissue culture medium containing 2 mM antibiotic antimycotic solution (Sigma-Aldrich, USA) and 2 mM L-glutamine (Sigma-Aldrich, USA; Cat no G7513). The mitogens (phytohaemagglutinin (PHA) (Thermo Scientific, USA; Cat no. 10576015) were used as a positive control stimulant in a final concentration of 5 μg/ml) and culture medium alone was kept as the negative control. The final volume of the culture was made to 200µl in triplicate wells.

The lyophilized *M. leprae* Cytosol Fraction (MLSA) (Catalog No. NR-19330; obtained from BEI resources under NIH leprosy research support contract) was reconstituted in sterile Phosphate-buffered saline (PBS) (pH 7.2) and added to the RPMI culture in 96-well tissue culture plates to achieve a final concentration of 10 μg/ml in the culture medium.

After that the culture plate was incubated at 37°C in 5% CO2 atmosphere, 150 µl culture supernatant was collected from each well after 42 hrs. of incubation and subsequently tested for cytokine assays for estimation of IFN-γ (R&D Systems, USA; Cat no. DY285B), IL-10 (R&D Systems, USA; Cat no. DY217B) and IL-12 (R&D Systems, USA; Cat no. DY1270) by ELISA in triplicate wells.

### The ELISA protocol for estimation of IFN gamma, IL-10 and IL-12

#### ELISA plate coating with capture antibody

The capture antibody was diluted with 1X PBS to make a working concentration of antibodies. Flat-bottom F96 MaxiSorp Nunc-Immuno Plates (Cat. No. 442404; Nunc, Denmark) 96-well microtiter plates were coated immediately with the 100 µl of capture antibodies of Interferon- either gamma (IFN-γ) or Interleukin-10 (IL-10) or Interleukin-12 (IL-12) using R&D Systems kits. After sealing with a sterilized sealer, the plates were incubated overnight at room temperature (RT). After incubation, the ELISA plates were washed thrice with 300 µl wash buffer (0.05% Tween 20 in PBS). After the washing step, the residual wash buffer was decanted by inverting and tapping the plate against clean blotting sheets. Later, the wells were blocked by adding a reagent diluent (RD) (1% BSA in PBS) (300 µl/well) and kept for incubation at RT for 60 min. The washing was done, and the ELISA plate was washed again with 300 µl wash buffer. These antibody-sensitized plates were used for ELISA assays.

### ELISA assay procedure

The standard and whole blood culture supernatant samples were diluted in Reagent Diluent (RD) (1% BSA in PBS), and 100 µl each of the culture supernatant was distributed into each well and after gentle tapping the plates were covered with sealer and incubated, at RT for 2 hrs. After the incubation, the washing was done three times with a wash buffer. Detection antibody (100 µl) diluted in RD was added to each well, and the ELISA plates were incubated at RT for 2 hrs. Later, the plates were washed three times with a wash buffer. After washing the plate, 100 µl streptavidin-HRP (1:40 dilution) was added to each well, and an ELISA plate was incubated at RT for 20 minutes in the dark to prevent direct exposure to light. The washing step was repeated thrice, and 100µl Substrate Solution (1:1 mixture of Color reagent A (H2O2) and Color reagent B (Tetramethylbenzidine) (Cat no. DY999, R&D Systems, USA) was added to each well. The plates were incubated at RT for 20 minutes in the dark without exposure to direct light. Fifty µl of stop solution (2N HCl) was added to each well of the plates to stop the reaction. The optical densities of each well were determined in a microplate reader at 450 nm. using Multiskan FC ELISA Reader (Thermo Fisher Scientific, USA).

### Software and statistical analysis

All cases of leprosy, along with their HHCs, were systematically analysed to determine any associations with the STH-helminthic positive and STH-helminthic negative groups by using the Odds ratio (OR) and relative risk ratio (RR). The characteristics and observations of the subjects were assessed using the Chi-square test. A p-value of less than 0.05 was deemed statistically significant, whereas a p-value exceeding 0.05 was considered not significant. ELISA data were expressed as mean ± SD in the graph, and the outliers of ELISA were removed by the ROUT method. The differences between optical density (OD) values obtained in the WBA culture of STH-helminthic positive and STH-helminthic negative of the leprosy patients and HHCs were determined using Mann-Whitney U test. Data were analysed using GraphPad Prism software version 5.0 (GraphPad Prism, La Jolla, CA).

## Results

### Study population characteristics

Three hundred sixty individuals participated in a study conducted in two leprosy-endemic districts: Purulia (n = 207) in West Bengal and Champa (n = 153) in Chhattisgarh. The study population included clinically confirmed index leprosy cases and their household contacts (HHCs). The analysis focused on key demographic factors, such as age, sex distribution, clinical classification (PB or MB), and the prevalence of STH infections.

### Demographic and clinical characteristics

#### Purulia district, west Bengal

In the study involving 60 index cases, 48 participants were male, with a mean age of 34.4 ± 14.7 years, while 12 participants were female, with a mean age of 35.1 ± 14.9 years. The predominant form observed was MB leprosy, which affected 96% of males and 92% of females. The odds ratio for MB leprosy among the male population was calculated to be 2.09(95% CI: 0.17–26.4), demonstrating a statistically significant association (p = 0.0001).

Furthermore, an analysis of 147 household contacts revealed that 62 were male (mean age: 29.3 ± 14.7 years) and 85 were female (mean age: 29.3 ± 15.2 years). Within this cohort, low frequencies of both PB and MB leprosy were detected. Specifically, among the male contacts, there were 3 cases of PB (5%) and 5 cases of MB (8%), whereas the female contacts exhibited 4 cases of PB (5%) and 2 cases of MB (2%) after follow-up of 5 years. (**Table 1**).

#### Champa district (Chhattisgarh)

In a detailed investigation involving 36 index cases, the demographic breakdown revealed that 29 participants were male, with a mean age of 34.4 years (± 14.7), while 7 participants were female, with a mean age of 30.9 years (± 15.2). - Remarkably, all male cases were classified as having MB leprosy, representing 100% of the male cohort. In contrast, among the female cases, 71% were diagnosed with MB leprosy, whereas 29% presented with PB leprosy. - The calculated odds ratio for MB leprosy among males was 26.82 (95% CI: 1.12 – 644.44), indicating a statistically significant association (p = 0.0005). Additionally, an assessment of 117 household contacts revealed that 54 were male, with a mean age of 29.6 years (± 15.4), and 63 were female, with a mean age of 29.1 years (± 15.2). - Within this group, eight males (15%) and five females (8%) were diagnosed with MB leprosy after follow-up of 5 years. - Furthermore, PB leprosy was observed in 3 females (5%), with no cases reported among the male contacts. (**Table 1**)

##### Microscopic identification of STH parasites in stool samples

In the study population (n=360), STH infections were identified in 26% of individuals diagnosed with leprosy and 17% of their HHCs. The odds ratio for STH infection among leprosy cases, compared to that among HHCs, was calculated to be 1.71 (95% CI: 0.98 – 2.98). Although these findings indicate a higher prevalence of STH infections among individuals with leprosy, the association did not reach statistical significance (p = 0.0703). Furthermore, we stratified our analysis by sex-specific patterns in STH co-infection among leprosy cases and their HHCs. Among males, 26% of index cases tested positive for helminths, whereas only 12% of male HHCs demonstrated the same. This discrepancy was statistically significant, yielding an odds ratio (OR) of 2.60 (95% CI: 1.22–5.55); p = 0.019, indicating that male index cases were significantly more likely to be co-infected with helminths. Conversely, among females, the prevalence of STH infection was 25% in index cases and 21% among HHCs contacts. This difference did not achieve statistical significance (OR = 1.26; 95% CI: 0.42–3.73; p = 0.772), suggesting no meaningful association within the female population. These findings indicate a potential sex-specific interaction between STH infections and leprosy status, with male cases exhibiting a notably higher likelihood of co-infection. (**Table 2**)

**Table 2:** Helminthic infection and its association with leprosy.

Subject	Total samples	STH Positive	STH Negative	p-value	OR (95% CI)
Leprosy Cases	96	25 (26%)	71 (74%)	0.070	1.71 (0.98 - 2.98)
HHCs	264	46 (17%)	218 (83%)		
Group	Sex	STH Positive	STH Negative	p-value	OR (95% CI)
Index cases	Male	20 (26%)	56 (74%)	0.019*	2.60 (1.22-5.55)
HHCs		14 (12%)	102 (88%)		
Index cases	Female	5 (25%)	15 (75%)	0.772	1.26 (0.42-3.73)
HHCs		31 (21%)	117 (79%)		

*Statistically significant at p < 0.05.

### Prevalence and distribution of intestinal parasites among study participants

We collected 360 stool samples from leprosy cases and their HHCs. We identified a significant prevalence of 128 intestinal STH parasitic infections. (**Table 3**) STHs were identified in 55% of the samples (n = 70), indicating a notable occurrence of helminthic co-infections within the study population. (**Table 2**) Among the identified helminths, hookworm species, specifically *Ancylostoma duodenale* and *Necator americanus*, were the most prevalent, detected in 30% of the samples (n = 38). Roundworms, represented by *Strongyloides stercoralis* larvae, *Ascaris lumbricoides*, and other nematodes, were observed in 16% of the samples (n = 20). The presence of tapeworms, such as *Taenia saginata* and *Hymenolepis nana*, was recorded in 3% of the samples (n = 4), while pinworms (*Enterobius vermicularis*) were detected in only 0.8% of the samples (n = 1). Furthermore, general ova and parasite (O&P) positivity was noted in 5% of the samples (n = 7), suggesting a non-specific helminthic infection. In addition to helminths, protozoan infections were also common. *Giardia lamblia* was detected in 12% of the samples (n = 16), and *Entamoeba histolytica* was found in 16% (n = 20). Additionally, the presence of bacteria, particularly *Escherichia coli*, was observed in 17.2% of the samples (n = 22). (**Table 3**).

**Table 3:** Soil-transmitted helminthic parasites detected in stool samples.

Name of the STH	Name of the STH species (intestinal parasite)	Total number (%)
Hookworm	*Ancylostoma duodenale, Necator americanus*	38 (30%)
Roundworm	*Strongyloides stercoralis larva, Ascaris lumbricoides, Nematoda*	20 (16%)
Ova & Parasite	*O&P*	7 (5%)
Pinworm	*Enterobius vermicularis*	1 (0.8%)
Tapeworm	*Taenia saginata Hymenolepis nana*	4 (3%)
Protozoan	*Giardia lamblia*	16 (12%)
	*Entamoeba histolytica*	20 (16%)
Bacteria	*E. coli*	22 (17.2%)
Total number of stool samples		128

### Leprosy families-household contacts follow-up

During the study, all HHCs were monitored every six months for the appearance of signs and symptoms of leprosy for over five years. Among the HHCs infected with STH, 13% (6 out of 46) developed leprosy. In comparison, among HHCs who tested negative for STH infection, 11% (24 out of 218) developed leprosy. The calculated RR was 1.18, indicating a marginally increased likelihood of leprosy among STH-positive contacts. However, this association did not attain statistical significance (p = 0.816), suggesting that STH infection may not represent a major independent risk factor for developing leprosy within this cohort (refer to **Table 4**).

**Table 4:** Overall Helminthic Burden and Disease Development.

Subject with STH infection status	Total Samples	Developed leprosy disease	Not developed leprosy disease	Chi- square tests	Relative Risk (RR)
HHC positive for STH infection	46	6 (13%)	40 (87%)	0.816	1.18
HHC Negative for STH infection	218	24 (11%)	194 (89%)		
Total	264	30 (11%)	234 (89%)		

### ELISA assay for cytokine IFN-γ, IL-12 and IL-10:

**Figure 1:**
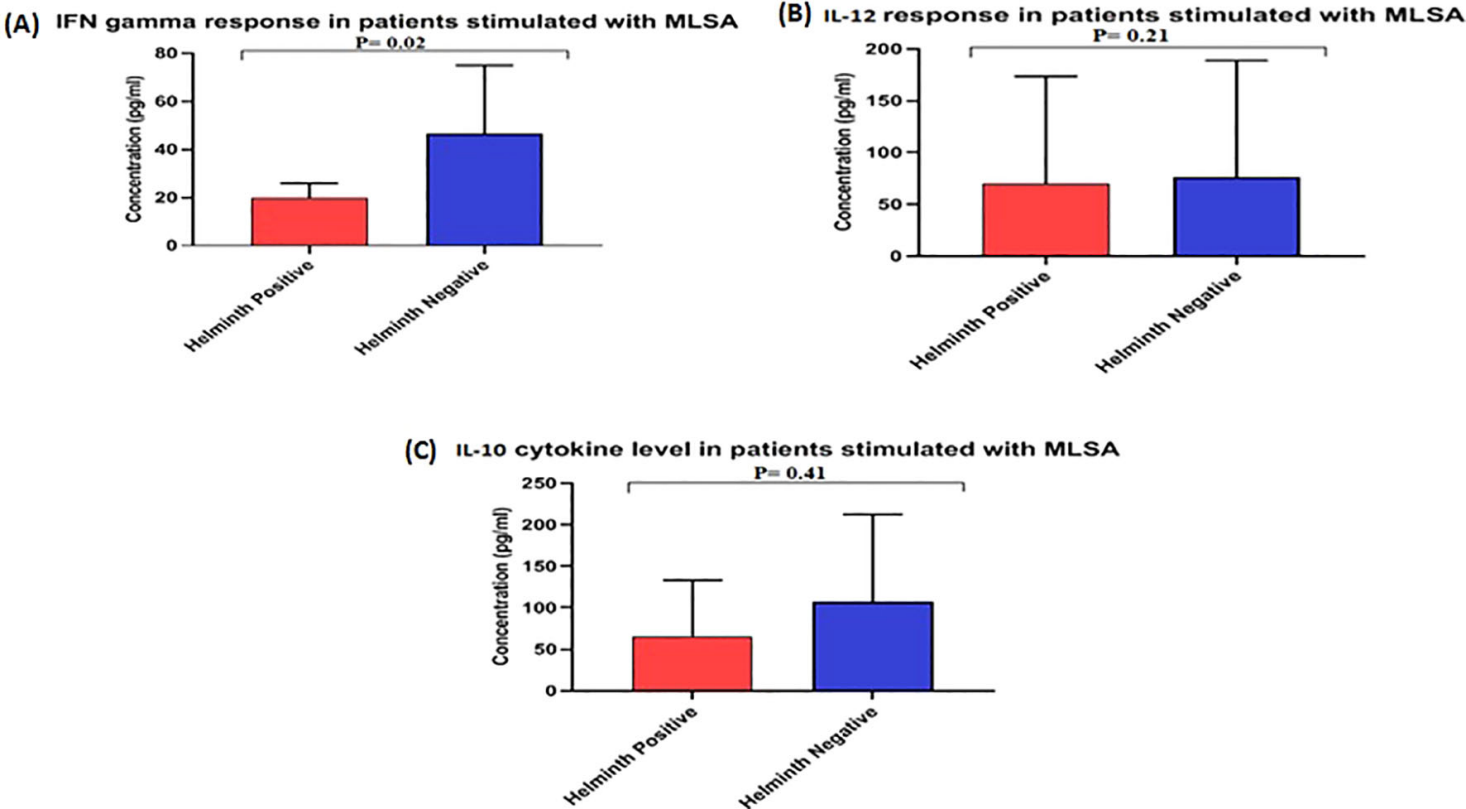
Level of cytokines **(A)** IFN γ, **(B)** IL12, and **(C)** IL10 in the culture supernatant of Whole Blood Assay stimulated with MLSA. Each bar represents mean concentration (pg/mL) with SD across each group. The OD was obtained at 450 nm. Manns Witney U test was performed for statistical analysis.

The statistical analysis of the cytokine levels showed that the leprosy group without STH infection had significantly higher levels of IFN-γ (P < 0.02) than that of the STH positive group, indicating a stronger Th1 immune response STH negative leprosy group. The mean IFN-γ value for STH-positive leprosy cases was 19.70 pg./ml, compared to 46.60 pg./ml for STH -negative leprosy cases (**Figure 1A**). This significant difference suggests that leprosy STH -negative have elevated levels of IFN-γ expressing Th1 immunity without STH infection. However, regarding IL-12 cytokine levels, the mean levels for STH -positive leprosy cases in the STH -negative group were almost equal, 70.22 and 76.46 pg./ml, respectively. (p=0.21) (**Figure 1B**).

The mean values for IL-10 cytokine in a leprosy patient’s STH -positive and negative groups were 66.03 pg./ml and 76.46 pg./ml, respectively (**Figure 1C**), showing no significant (p>0.41) difference between the groups.

## Discussion

Neglected tropical diseases (NTDs), such as leprosy and STH, exhibit high prevalence rates in Southeast Asia, particularly in India, where they disproportionately affect vulnerable and underserved populations. A substantial body of research indicates that STH infections may modulate host immunity by shifting the protective Th1 responses toward a Th2-dominant immune profile, thereby increasing susceptibility to leprosy (8, 19, 20). Within this framework, our research sought to investigate the parasitological burden and immunological profiles of leprosy patients and their HHCs in the endemic regions of Chhattisgarh and West Bengal. We specifically examined the influence of STH co-infection on disease susceptibility and immune response. This study contributes to the accumulating evidence that STH co-infections can significantly alter the immune landscape in leprosy, with potential implications for disease progression and transmission. These findings may inform public health policies in Southeast Asia, facilitating the development of targeted interventions addressing both leprosy and STH infections.

The prevalence of STH infections among cases of leprosy was found to be 26%, higher than the 17% observed among HHCs. The most prevalent STH species identified included hookworms (*Ancylostoma duodenale and Necator americanus*) and roundworms (*Ascaris lumbricoides*). (**Table 3**) A statistically significant association was established among male index cases, revealing that STH-positive individuals had 2.6 times greater odds (OR: 2.60; 95% CI: 1.22–5.55; p = 0.019) (**Table 2**) of developing MB leprosy compared to their STH-negative counterparts. These findings corroborate earlier research conducted by Oktaria et al. (19), which reported a higher prevalence of STH infections among individuals with leprosy in Indonesia. This suggests a potential immunomodulatory effect of chronic STH exposure, which may augment susceptibility to *M. leprae* infection. Additionally, Dennison et al. (9) identified helminth co-infection as one of several potential risk factors associated with leprosy, particularly in conjunction with micronutrient deficiencies, such as vitamin D. While the overall comparison between cases and HHCs did not reach statistical significance (OR = 1.71, p = 0.07), the sex-stratified results indicate the presence of specific subpopulations wherein the co-infection of STH and leprosy may be more pronounced.

The significant association observed in males in our study may indicate biological or behavioural differences that influence STH exposure or immune responses. However, the lack of statistical significance in the overall comparison and among females suggests that STH infection is likely one of several interacting risk factors contributing to susceptibility. This underscores the need for further research to fully understand the complex dynamics of leprosy susceptibility and the role of STH infections.

This study examined the’ demographic and clinical characteristics of index leprosy cases and their HHCS in two endemic districts: Purulia (West Bengal) and Champa (Chhattisgarh). (**Table 1**) The findings revealed a predominance of MB leprosy among male index cases. In Purulia, males were over twice as likely to present with MB disease compared to females (odds ratio [OR] = 2.09; 95% confidence interval [CI]: 0.17–26.4), while in Champa, this likelihood was significantly higher (OR = 26.82; 95% CI: 1.12–644.44). These findings necessitate cautious interpretation due to the wide confidence intervals, particularly in Champa, which may result from a limited number of PB cases and an overall restricted sample size.

The male predominance in MB cases aligns with various epidemiological reports indicating a higher risk among males worldwide. Biological factors, including sex hormone modulation of immune responses, may contribute to this difference. Testosterone is linked to a downregulation of cell-mediated immunity, potentially leading to higher bacterial loads and chronic conditions in males, whereas estrogen may enhance protective immune responses (21, 22). Additionally, societal factors such as occupational exposure and delayed treatment-seeking behaviours may further increase male vulnerability to MB disease progression (23).

The age distribution among index cases and their HHCs indicates ongoing transmission of leprosy primarily within younger and economically active age groups. This highlights the critical importance of early detection and preventive strategies at the household and community levels. The urgency and significance of these measures in controlling the spread of leprosy are apparent, highlighting the need for proactive public health interventions.

The absence of PB cases among male index patients in Champa is significant and may suggest under-detection or diagnostic biases. This underscores the need for more detailed community-level studies, including skin smears and molecular diagnostics, to better characterize the spectrum of disease. The findings also highlight the necessity of integrating gender-sensitive approaches into leprosy control programs. Active case detection should specifically target men who may delay seeking care, and communication strategies tailored to community gender dynamics could enhance early diagnosis and reduce transmission. This is particularly relevant in Southeast Asia, where the burden of leprosy and STH infections remains high, and our research can significantly contribute to the development of such gender-sensitive approaches.

While our study provides significant insights into the relationship between STH infections and leprosy susceptibility, it is with limitations. The relatively small sample size, especially among female cases, may have impacted the precision of odds ratio estimates. Therefore, future research with larger cohorts and longitudinal follow-ups must clarify sex-specific risks and disease progression dynamics. Additionally, molecular typing and immune profiling could provide deeper insights into host-pathogen interactions. These findings should be interpreted within this context; further investigation is warranted to confirm and expand upon these results.

This study’s primary objective was to understand STH’s role in susceptibility to leprosy comprehensively. Our findings indicate that STH infections induce a Th2-biased immune response, suppressing Th1-driven cytokines, including IFN-gamma. This cytokine is essential for the activation of macrophages and for the effective control of intracellular pathogens (24). The cytokine profiling conducted via ELISA further substantiates this immunological shift. The levels of IFN-γ, a Th1 cytokine critical in managing *M. leprae*, were significantly lower in STH-positive leprosy cases (mean: 19.70 pg/ml) compared to STH-negative cases (mean: 46.60 pg/ml; p < 0.02). This observation suggests that STH infection suppresses protective immune responses, supporting earlier findings that Th2 responses induced by STH can inhibit Th1-mediated immunity. Such suppression may impair the host’s ability to effectively control *M. leprae* infection and increase the likelihood of progressing to the MB form, which has also been noted in cases involving *M. tuberculosis* (25). These results resonate with studies from Brazil (9, 10) and Indonesia (19), which documented reduced IFN-γ levels and a heightened susceptibility in STH co-infected leprosy patients, thereby underscoring the significance of understanding immune responses in the context of disease susceptibility. Conversely, the levels of IL-12 did not demonstrate a significant difference between the groups (mean: 70.22 vs. 76.46 pg/mL, p = 0.21). This finding suggests that the initial induction of Th1 pathways may remain intact; however, the downstream effector functions, particularly IFN-γ production, appear to be selectively suppressed. Furthermore, IL-10 levels, which indicate regulatory T cell activity, did not significantly differ (66.03 vs. 76.46 pg/mL; p > 0.41), indicating that STH infection might not play role in the induction of TH2 type of immunity. Further, this suggests that the primary effect may be concentrated on downstream Th1 effector functions rather than on upstream induction or broad immunoregulation. These immunological findings reflect those Dennison et al. (9) reported in southeastern Brazil, where helminth co-infection and vitamin D deficiency were associated with an increased risk of MB leprosy. Similarly, our findings emphasise that co-infections can hinder the host’s capacity to mount an adequate Th1 response, potentially altering the disease outcomes.

Furthermore, ecological parallels exist with the study by Oktaria et al. (19) in Indonesia, which identified a significant association between STH infections and leprosy in households where both conditions co-occurred. Although the cross-sectional design limited conclusions about temporality or causality, the trend toward a higher incidence of disease among STH-positive HHCs (relative risk: 1.18) highlights the need for long-term follow-up studies to evaluate progression risk and immune modulation. This hypothesis is further supported by Diniz et al. (10), who reported that STH inhibited Th1 cytokine responses, such as IFN-γ, in tuberculoid leprosy patients, potentially leading to an MB phenotype. Our findings align with this concept, as the higher incidence of MB leprosy among STH-positive individuals suggests an immunological shift influenced by STH-induced suppression of protective Th1 responses.

The consistent presence of polyparasitism, including protozoa such as *Giardia lamblia* and *Entamoeba histolytica*, alongside bacteria like *Escherichia coli* and helminths, suggests a significant dysregulation of the gut microbiome that may affect immune function. Although these preliminary findings open avenues for investigating the gut-skin-immune axis in leprosy pathogenesis, caution is warranted due to the lack of a corresponding statistical association at the population level.

The detection of STH parasites relied on conventional microscopic techniques with limited sensitivity, potentially underestimating true STH prevalence. The relatively small sample size and failure to adjust for confounding factors like age, nutritional status, and socioeconomic conditions may also limit nuanced associations. Additionally, our focus on three cytokines (IFN-γ, IL-12, and IL-10) restricts understanding of the broader cytokine network involved in leprosy-STH interactions. Future research should include a broader cytokine panel and gut microbiome profiling for more comprehensive insights.

This study provides novel data from two understudied endemic districts in India—Purulia (West Bengal) and Champa (Chhattisgarh)—adding important geographic and ecological context to the literature. The sex-disaggregated analysis presents a nuanced understanding of how STH co-infection may vary by gender, a factor not thoroughly explored in earlier studies. Our findings suggest that microbial ecology might play a significant role in modulating disease risk and transmission by identifying a broad spectrum of STH, protozoa, and bacterial pathogens.

In summary, this study compellingly reinforces the hypothesis that STH infections are not merely incidental in leprosy-endemic regions but also significant modulators of host immunity. The Th1-suppressive effects of STHs—evidenced by reduced IFN-γ levels—may impair effective immune responses to *M. leprae*, potentially increasing susceptibility and hindering bacterial clearance. These findings highlight a critical intersection between STH parasitic co-infections and leprosy pathogenesis, posing challenges to achieving zero transmission. It is essential to adopt integrated approaches to break the leprosy transmission chain and support the goals of the WHO Global Leprosy Strategy and India’s National Leprosy Eradication Programme (NLEP). We recommend incorporating routine deworming, WASH (Water, Sanitation, and Hygiene) improvements, and nutritional support into leprosy surveillance and case management frameworks. These strategies can bolster host immunity, reduce co-infection burdens, and strengthen the impact of existing control programs. Further longitudinal and mechanistic research will be crucial in identifying reliable immunological and parasitological markers for early risk stratification and targeted intervention.

## Data Availability

The original contributions presented in the study are included in the article/supplementary material. Further inquiries can be directed to the corresponding author.

## References

[1] Irgens LM. Oppdagelsen av leprabasillen [The discovery of leprosy bacillus. Tidsskr Nor Laegeforen. (2002) 122:708–9. Norwegian. 11998735

[2] Han XY Seo YH Sizer KC Schoberle T May GS Spencer JS. A new Mycobacterium species causing diffuse lepromatous leprosy. Am J Clin Pathol. (2008) 130:856–64. doi: 10.1309/AJCPP72FJZZRRVMM 19019760

[3] Dhillon GP. NLEP–current situation and strategy during the 11th plan period (2007-2012). J Indian Med Assoc. (2006) 104:671–2. 17474280

[4] World Health Organisation. Weekly Epidemiological Record (2023). WER9837-eng-fre.pdf (who. int).

[5] Jain SK Dwivedi A Shrivastava A Pavadai V Vidyardhini R Venkatesh S. Prevalence of soil transmitted helminthic infections in India in current scenario: A systematic review. J Commun Dis. (2016) 48:24–35.

[6] Soil-transmitted helminth infections(2024). who. int.

[7] Yasmin H Varghese PM Bhakta S Kishore U. Pathogenesis and host immune response in leprosy. Adv Exp Med Biol. (2021) 1313:155–77. doi: 10.1007/978-3-030-67452-6_8 34661895

[8] Diniz LM Magalhães EF Pereira FE Dietze R Ribeiro-Rodrigues R. Presence of intestinal helminths decreases T helper type 1 response in tuberculoid leprosy patients and may increase the risk for multi-bacillary leprosy. Clin Exp Immunol. (2010) 161:142–50. doi: 10.1111/j.1365-2249.2010.04164.x PMC2940159 20491787

[9] Dennison CL de Oliveira LB Fraga LAO E Lima RS Ferreira JA Clennon JA. Mycobacterium leprae-helminth co-infections and vitamin D deficiency as potential risk factors for leprosy: A case-control study in south-eastern Brazil. Int J Infect Dis. (2021) 105:261–6. doi: 10.1016/j.ijid.2021.02.048 33592342

[10] Diniz LM Zandonade E Dietze R Pereira FE Ribeiro-Rodrigues R. Short report: Do intestinal nematodes increase the risk for multibacillary leprosy? Am J Trop Med Hyg. (2001) 65:852–4. doi: 10.4269/ajtmh.2001.65.852 11791986

[11] Pearlman E Kazura JW Hazlett FE Jr Boom WH. Modulation of murine cytokine responses to mycobacterial antigens by helminth-induced T helper 2 cell responses. J Immunol. (1993) 151:4857–64. doi: 10.4049/jimmunol.151.9.4857 8409444

[12] Ferreira AP Faquim ES Abrahamson IA Macedo MS. Immunization with Ascaris suum extract impairs T cell functions in mice. Cell Immunol. (1995) 162:202–10. doi: 10.1006/cimm.1995.1070 7743547

[13] Macedo MS Faquim-Mauro E Ferreira AP Abrahamsohn IA. Immunomodulation induced by Ascaris suum extract in mice: effect of anti-interleukin-4 and anti-interleukin-10 antibodies. Scand J Immunol. (1998) 47:10–8. doi: 10.1046/j.1365-3083.1998.00251.x 9467652

[14] Hagge DA Parajuli P Kunwar CB Rana DRSJB Thapa R Neupane KD. Opening a can of worms: leprosy reactions and complicit soil-transmitted helminths. EBioMedicine. (2017) 23:119–24. doi: 10.1016/j.ebiom.2017.08.026 PMC5605364 28882756

[15] Chopra P Shekhar S Dagar VK Pandey S. Prevalence and risk factors of soil-transmitted helminthic infections in the pediatric population in India: A systematic review and meta-analysis. J Lab Physicians. (2022) 15:4–19. doi: 10.1055/s-0042-1751319 37064993 PMC10104723

[16] Turankar RP Singh V Gupta H Pathak VK Ahuja M Singh I. Association of non-tuberculous mycobacteria with Mycobacterium leprae in an environment of leprosy endemic regions in India. Infect Genet Evol. (2019) 72:191–8. doi: 10.1016/j.meegid.2018.11.010 30445113

[17] Kotgire S. Microbiological stool examination: Overview. J Clin Diagn Res. (2012) 6:503–9.

[18] Tran TAT Grievink HW Lipinska K Kluft C Burggraaf J Moerland M. Whole blood assay as a model for *in vitro* evaluation of inflammasome activation and subsequent caspase-mediated interleukin-1 beta release. PloS One. (2019) 14:e0214999. doi: 10.1371/journal.pone.0214999 30958862 PMC6453527

[19] Oktaria S Effendi EH Indriatmi W van Hees CL Thio HB Sjamsoe-Daili ES. Soil-transmitted helminth infections and leprosy: a cross-sectional study of the association between two major neglected tropical diseases in Indonesia. BMC Infect Dis. (2016) 16:258. doi: 10.1186/s12879-016-1593-0 27278453 PMC4898373

[20] Fróes LAR Jr Toma TS Jachiet M Rousset L Poderoso RE Trindade MAB. Bacterial, fungal and parasitic co-infections in leprosy: A scoping review. PloS Negl Trop Dis. (2023) 17:e0011334. doi: 10.1371/journal.pntd.0011334 37216331 PMC10202305

[21] da Silva LM Dias CM Foss NT. Gender differences in leprosy: A review of epidemiological and immunological aspects. Rev da Sociedade Bras Medicina Trop. (2018) 51:149–56. doi: 10.1590/0037-8682-0471-2017

[22] Fritsche AR Silva BJA Jardim MR. Immune response and gender in leprosy: What do we know? Expert Rev Anti-infective Ther. (2020) 18:1041–9. doi: 10.1080/14787210.2020.1827382

[23] Pescarini JM Strina A Nery JS Skalinski LM Andrade K Penna MLF. Socioeconomic risk markers of leprosy in high burden countries: A systematic review and meta analysis. PLoS Negl Trop Dis. (2018) 12:e0006622. doi: 10.1371/journal.pntd.0006622 29985930 PMC6053250

[24] Kiflie A Bewket G Tajebe F Abate E Schön T Blomgran R. Helminth species-specific effects on IFN-γ producing T cells during active and latent tuberculosis. PLoS Negl Trop Dis. (2023) 17:e0011094. doi: 10.1371/journal.pntd.0011094 36662839 PMC9891516

[25] Resende Co T Hirsch CS Toossi Z Dietze R Ribeiro-Rodrigues R. Intestinal helminth co-infection has a negative impact on both anti-Mycobacterium tuberculosis immunity and clinical response to tuberculosis therapy. Clin Exp Immunol. (2007) 147:45–52. doi: 10.1111/j.1365-2249.2006.03247.x 17177962 PMC1810442

